# Development and validation of automatic tools for interactive recurrence analysis in radiation therapy: optimization of treatment algorithms for locally advanced pancreatic cancer

**DOI:** 10.1186/1748-717X-8-138

**Published:** 2013-06-07

**Authors:** Kerstin A Kessel, Daniel Habermehl, Andreas Jäger, Ralf O Floca, Lanlan Zhang, Rolf Bendl, Jürgen Debus, Stephanie E Combs

**Affiliations:** 1 Department of Radiation Oncology, Heidelberg University Hospital, Im Neuenheimer Feld 400, Heidelberg, 69120, Germany; 2 German Cancer Research Center (dkfz), Im Neuenheimer Feld 280, Heidelberg, 69120, Germany; 3 Department of Medical Informatics, Heilbronn University, Max-Planck-Str. 39, Heilbronn, 74081, Germany

**Keywords:** Pancreatic cancer, Recurrence analysis, Electronic data processing

## Abstract

**Background:**

In radiation oncology recurrence analysis is an important part in the evaluation process and clinical quality assurance of treatment concepts. With the example of 9 patients with locally advanced pancreatic cancer we developed and validated interactive analysis tools to support the evaluation workflow.

**Methods:**

After an automatic registration of the radiation planning CTs with the follow-up images, the recurrence volumes are segmented manually. Based on these volumes the DVH (dose volume histogram) statistic is calculated, followed by the determination of the dose applied to the region of recurrence and the distance between the boost and recurrence volume. We calculated the percentage of the recurrence volume within the 80%-isodose volume and compared it to the location of the recurrence within the boost volume, boost + 1 cm, boost + 1.5 cm and boost + 2 cm volumes.

**Results:**

Recurrence analysis of 9 patients demonstrated that all recurrences except one occurred within the defined GTV/boost volume; one recurrence developed beyond the field border/outfield. With the defined distance volumes in relation to the recurrences, we could show that 7 recurrent lesions were within the 2 cm radius of the primary tumor. Two large recurrences extended beyond the 2 cm, however, this might be due to very rapid growth and/or late detection of the tumor progression.

**Conclusion:**

The main goal of using automatic analysis tools is to reduce time and effort conducting clinical analyses. We showed a first approach and use of a semi-automated workflow for recurrence analysis, which will be continuously optimized. In conclusion, despite the limitations of the automatic calculations we contributed to in-house optimization of subsequent study concepts based on an improved and validated target volume definition.

## Background

In radiation oncology recurrence analysis is an important part in the evaluation process and clinical quality assurance of treatment concepts. It can provide useful information for subsequent dose-escalation strategies, adaptation of target volume definition, requiring higher radiation doses, or identification of areas at high-risk possibly amenable to surgical resection. However, it involves handling a variety of significant datasets from numerous information systems in different documentation styles. Especially when analyzing a large number of patients, it can be immensely time consuming [1,2]. The ultimate goal is the correlation of pre-treatment imaging, radiation treatment plans and follow-up imaging with less effort, even in centers where multiple treatment planning systems are in use. To date, no system provides the base to allow such analyses with one mouse click. As a first step, we generated a common database summing all patient data from treatment planning, radiation plans and follow-up including imaging, lab data and clinical evaluation. All information within this system can be kept together to facilitate clinical analyses. To correlate imaging and treatment planning within this system, to date, manual matching and evaluation is required. Therefore, to support this process we adopted several tools to accelerate the evaluation workflow for a treatment planning and recurrence analysis. These tools were integrated into the designed database workflow, and validated on the example of patients with locally advanced pancreatic cancer treated with radio-chemotherapy.

For pancreatic cancer it is known that about 30-40% of patients are locally advanced and therefore not curatively resectable [3]; therefore, strategies for downsizing are in focus using different chemotherapeutic combinations or chemoradiation. In line with several analyses we could show previously that radiation and chemotherapy may lead to downsizing and secondary resection in about 30-40% of the patients [4]. However, much controversy exists about target volume definition, from centers involving large areas of lymphatic spread into the clinical target volume (CTV) to strategies focusing on the gross tumor volume (GTV) only [3,5,6]. Optimizing treatment volumes can be performed based on recurrence and volume analysis, therefore, this clinical case was taken to evaluate the connected tools for recurrence and matching analysis.

Patterns of tumor progression can be determined by calculating the distance of the recurrence from the primary tumor, and the location of the recurrence in relation to the target volumes and isodose volumes. Both factors provide feedback on the question about adequate target volume definition and safety margins for radiation therapy. With respect to the emerging radiation techniques, e.g. stereotactic body radiation therapy (SBRT) or particle therapy, to be more precisely than conventional photon techniques, the decision on how much tumor-surrounding normal tissue should be irradiated becomes more important. Besides, with the possibility of a high precise irradiation, higher doses can be applied with the same radiation exposure for organs at risk (OAR) and normal tissue.

Measuring the distance of the recurrence gives information about where to expect the majority of recurrences in general and consequently to what extent elective regions at risk for subclinical tumor spread should be added to the safety margin or CTV. Planning target volumes (PTV) are added depending on the treatment technique, overall setup and patient positioning.

The aim of this evaluation is to implement and connect interactive analysis tools to support the analysis workflow [7] and ultimately optimize patient treatment through validation in a cohort of patients with locally advanced pancreatic cancer.

## Methods

For validation of the developed data tools we randomly chose 9 patients with locally advanced pancreatic cancer treated with neoadjuvant radio-chemotherapy with known progression after therapy, which developed local recurrence. These patients are a subgroup of all patients (n = 198) treated at the Department of Radiation Oncology at the University Hospital Heidelberg between 2003-2010 (see Habermehl et. al. [4]). In one patient RT was delivered as intensity-modulated radiation therapy (IMRT) and 8 patients had three-dimensional (3D) conformal radiation therapy. All 9 patients had regular follow-up CT imaging according to institutional guidelines [8]. Follow-up scans were performed with a contrast-enhanced multislice CT imaging in 30°-45° RAO (Right Anterior Oblique) positioning to reduce artifacts in the pancreatic head region [9].

For treatment planning, we acquired contrast-enhanced multislice CT-imaging, with a slice thickness of 3 mm or 5 mm and fused it within the treatment planning system. Target volume definition and treatment planning were performed using the 3D treatment planning software Oncentra MasterPlan (Nucletron, USA) or the IMRT planning systems KonRad (Siemens OCS, Germany).

We defined the gross tumor volume (GTV) as the macroscopically visible primary tumor. A clinical target volume (CTV) was defined including the GTV plus surrounding lymph nodes and areas of lymphatic spread adding about 2-3 cm in all directions; for the boost volume a margin of 0.5-1 cm was added to the GTV. A planning target volume (PTV) was added depending on the treatment machine and patient setup of 3-5 mm. A median total dose of 44.5 Gy to the PTV and 53.5 Gy to the boost in median single doses of 1.86 Gy was applied.

For the recurrence analysis all images as well as the radiation plans were gathered and stored in a documentation system [10] in addition to clinical patient data, e.g. basic patient information and treatment data.

The analysis workflow has been established as follows: after an automatic registration of the radiation planning CTs with the follow-up images, the recurrence volumes are segmented manually by a radiation oncologist. Based on these volumes the DVH (dose volume histogram) statistic is calculated, followed by the determination of the dose applied to the region of recurrence and the distance between the boost and recurrence volume.

The study is in compliance with the Helsinki Declaration (Sixth Revision, 2008). A vote by the independent Ethics Committee of the Medical Faculty, Heidelberg has been obtained (Ref.-Nr.: S-483/2011).

### Image registration

Image registration and analysis of the geometric variances is essential to determine the applied dose to the recurrence. We used the AVID (analysis of variations in interfractional radiotherapy) framework for calculating the geometric variances. The framework was created with the goal to automatically analyze geometric and dosimetric variances of large collections of patient data, based on MatchPoint [11]. AVID allows exchanging the registration method on demand. In the present evaluation, a widely accepted mutual-information-based rigid registration method [12] is used, taking translational and rotational changes of the moving images into account.

For our calculation, translational and rotational motions along the left-right, anterior-posterior and superior-inferior axes are determined with respect to the PTV and spinal cord segmented on the planning CT. For each patient *p* ∈ {1, …, *N*} the follow-up CTs cct_p_ ∈ {1, …, *N*} are registered to the original planning CT. As a result, each determined geometrical variance is represented by T_p,cct_ = (*tx, ty, tz, rpx, rpy, rpz, rx, ry, rz*). While the first three values describe the translational shifts, the values four to six specify the rotation point and the last three values the rotation angles.

### Segmentation

After registration the local pancreatic recurrence is manually segmented on the registered follow-up CT images. On each slice of the three-dimensional data cube, the recurrence area is delineated.

### Dose calculation

The AVID framework provides further dose analysis tools for calculating dosimetric variances of defined volumes of interest (VOI) from both the segmented structures on the planning CT and the manually segmented recurrence volume. AVID uses the RTToolbox [13] to determine the dosimetric variances - a dedicated dose analysis library developed at the German Cancer Research Center (Heidelberg, Germany) containing analysis routines for descriptive dose statistics parameters together with DVHs. To ensure a precise dose analysis, voxelization is done on several resolution levels, to ensure all calculations have subvoxel accuracy.

### Distance measuring & recurrence analysis

The automatic pre-registered CT images were revised and again manually registered with focus on the surrounding vessels (aorta, superior mesenteric artery, celiac trunc, portal vein, hepatic arteries) of the initial tumor mass, bone anatomy and location of organs at risk in close proximity. For validating the automatic calculations the boost volume was contoured by an experienced radiation oncologist; additionally, using a manual enlargement, the boost was expanded with circumferential margins of 1 cm, 1.5 cm and 2 cm. We correlated the region of the recurrence with the original treatment plan. Generally, for treatment planning, coverage of the PTV by the 95%-isodose is attempted according to ICRU criteria. Since pure “local” failure is difficult to distinguish from marginal recurrence in these patients, we defined the 80%-isodose as our cut-off line for recurrence description.

## Results

In our group of 9 patients we processed CT scans with a 20° up to a 60° rotation. Consequently, pre-initialization of the CT scans was part of the registration process.

**Figure 1:**
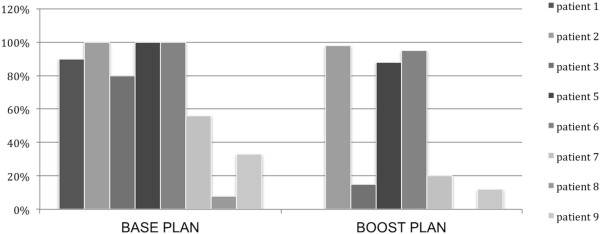
Diagram showing the percentage of the recurrence volume within the 80%-isodose volume of the base and boost plan, calculated by the AVID dose algorithm.

After segmentation of the local recurrence the dose evaluation was performed on follow-up CT scans. The initial dose distribution derived from the original treatment plan was correlated with the recurrence volume on the follow-up images. We calculated the DVH statistics of the 8 patients with 3D conformal radiation therapy for the base and boost plan separately (see Table 1). These plans were applied sequentially. We excluded the IMRT patient as the boost dose is calculated into one sum plan for radiation and we had no separate dose files available for evaluation. Figure 1 shows the percentage of the recurrence volume within the 80%-isodose volume.

**Table 1:** Dose statistic of the recurrence volume grouped separately for the base and boost plan (patient 4 with IMRT is excluded; patient 1 had no boost irradiation), calculated by the AVID dose algorithm

				**Base plan**				**Boost plan**			
**Patient**	**fx base**	**fx boost**	**Recurrence vol [ml]**	**Vol. in 80% isodose [ml]**	**Min [Gy]**	**Max [Gy]**	**Median [Gy]**	**Vol. in 80% isodose [ml]**	**Min [Gy]**	**Max [Gy]**	**Median [Gy]**
1	28	nd	79,31	71,08	31,02	53,15	51,17				
2	24	5	48,89	48,89	42,15	43,77	42,80	47,99	4,35	9,20	8,59
3	24	5	59,26	49,65	19,38	43,49	40,89	8,97	0,29	8,74	3,35
5	23	5	311,04	311,04	37,16	43,27	41,02	75,49	4,18	9,30	8,95
6	24	5	29,93	29,93	39,65	43,90	42,59	28,35	1,14	9,25	8,91
7	24	5	66,58	37,28	16,41	44,59	37,63	13,14	0,17	9,27	1,69
8	24	5	221,82	17,42	0,75	40,53	2,86	0,00	0,05	2,66	0,15
9	25	3	104,76	34,13	3,74	47,90	32,94	12,95	0,15	5,64	3,17

**Figure 2:**
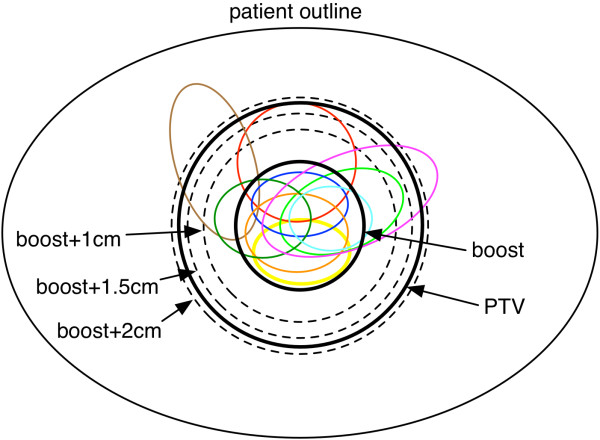
Schematic diagram on the location of the recurrence (colored structures) in the relation to the boost volume, boost + 1 cm, boost + 1.5 cm and boost + 2 cm volumes assessed during validation by the physician; the drawn PTV is arbitrary representing common clinical relations.

The measurement of the location of the recurrence within the boost volume, boost + 1 cm, boost + 1.5 cm and boost + 2 cm volumes is described schematically in Figure 2. Patient 9 had a biliary stent in the follow-up CT but not initially. This made segmentation difficult for the physician because of subsequent transposition of anatomic structures in comparison to the initial CT scan. Weight loss was clearly seen in all patients in the follow-up CTs. Particularly the mesenteric and subcutaneous fat tissue decreased, which cannot be taken into account very well by both the automatic algorithms and the physician during registration.

Patient 7, 8 and 9 had large recurrences growing within the boost volume/border and outfield up to 6.26 cm, 4.05 cm and 1.99 cm respectively (measured on one representative axial slide). This can also be seen in the calculated results in in Figure 1. Only 20%, 0% and 12% of the recurrence volume of patient 7, 8 and 9, respectively, lie within the 80%-isodose volume.

**Figure 3:**
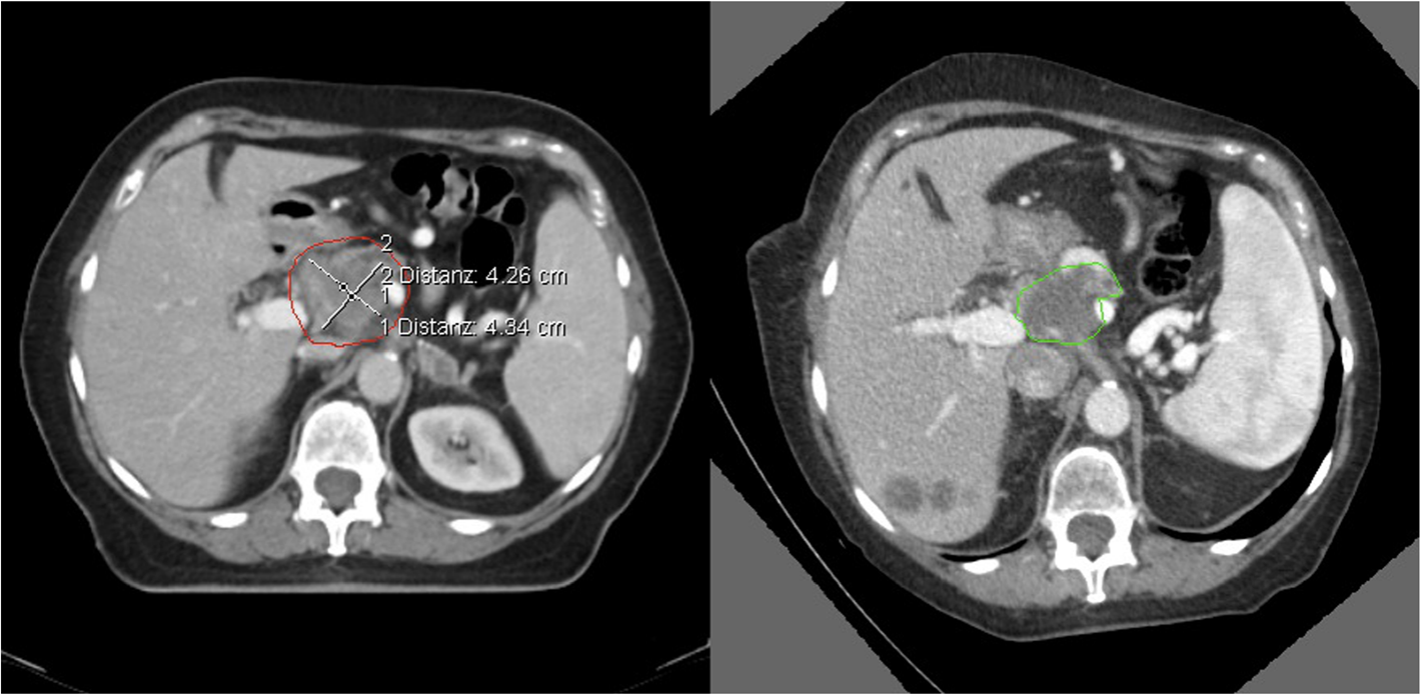
CT scans of patient 3; left: planning CT, right: follow-up CT after automatic registration with rotation and severe deformation.

Patient 3 had a severe deformation in the follow-up CT scan (see Figure 3) compared to the planning CT. That might be the reason for the different finding of the physician and automatic calculation. In the validation process, the physician found the recurrence to be within the boost and boost + 1 cm volume. However, the automatic algorithm calculated 84% in the PTV and only 15% in the boost 80%-isodose volume.

The validation showed a very well match for patient 1 and 2 as well as 5 and 6 (cp. Table 2). For patient 1 and 2 the recurrence laid in the PTV 90% and 100%, respectively. For patient 2 the algorithm calculated 98% match for the boost volume; patient 1 had no boost VOI delineated for calculation. Recurrences for patient 5 and 6 were in the boost volume and slightly outside (1 cm and 1.15 cm), which correlates with the validation results to be within the boost + 1 cm volume for patient 5 (algorithm 88% match); and within the boost + 1.5 cm volume for patient 6 (algorithm 95% match).

**Table 2:** Overview on the location of the recurrence in relation to the boost volume, boost + 1 cm, boost + 1.5 cm and boost + 2 cm volumes, delineated by the radiation oncologist

**Patient**	**In boost**	**In boost + 1 cm**	**In boost + 1.5 cm**	**In boost + 2 cm**
1	X			
2	X			
3	X	X		
4	X	X		
5	X	X		
6	X	X	X	
7	X	X	X	X
8	X	X	X	X
9	X	X	X	X

Recurrence analysis demonstrated that all recurrences except one occurred within the defined GTV/boost volume; one recurrence developed beyond the field border/outfield. With the defined distance volumes in relation to the recurrences, we could show that most recurrent lesions were within the 2 cm radius of the primary tumor (cp. Figure 2). Two large recurrences extended beyond the 2 cm, however, this might be due to very rapid growth and/or late detection of the tumor progression. Analysis of the spatial relationship of the recurrences proved the concept that extension of the CTV/PTV into the ventro-lateral regions of lymphatic spread of approximately 2 cm encloses the regions of tumor recurrence. No growth dorsally in the region of the boost was observed; therefore, no significant enlargement into this region seems required. Additionally, no difference between the lateral right-left sides could be shown.

## Discussion

The present analysis demonstrated that most recurrences of locally advanced pancreatic cancer develop within the 2 cm radius of the primary tumor, thus a CTV extension of 2 cm ventro-laterally is required to address recurrences by enclosing high-risk regions. Nevertheless, our study has several limitations mainly due to morphological changes of the abdominal anatomic structures and the CT scan setup. Pancreatic cancer patients are known to experience a significant loss of body weight because of the underlying disease and the multimodality treatment leading to a median loss of approximately 13% of visceral adipose tissue [14]. This in turn has direct impact on the topographic anatomy and relationship of abdominal organs such as liver, pancreas, stomach, intestine and kidneys.

Comparison and registration of different CT scans recorded over several months is not a simple task and a potential source of uncertainty. In our analysis, we performed a rigid registration, as others showed to be beneficial in pancreatic registration [15]. Nevertheless, we don’t know if this method is sufficient enough; comparison with an elastic registration should be explored. Furthermore, as described in the methods section, follow-up CT scans were acquired in a 30°-45° RAO position of the patient according to our in-house standard [8,9,16]. This leads to a transposition of intra-abdominal organs to the right side and affection of tumors adherent on mesenteric vessels. Even with careful manual registration of different CT datasets certain impreciseness cannot be avoided. However, an experienced radiation oncologist validated all correlations to certify correct matching and relation of boost and target volumes (cp. Table 2). This was the case in all patients evaluated. Therefore, manual validation could show overall excellent correlation with anatomical relations of the boost to the treated target volumes.

For pancreatic tumors, a dose-response-relationship has been shown in previous studies [17]. Together with the pattern of recurrence evaluated in the present manuscript as well as the data showing that all recurrences occurred infield within the 80%-isodose of the high-dose boost treatment, further extension of target volumes may not be of benefit. It even seems that smaller margins dorso-laterally of around 1 cm, and CTV extensions ventro-laterally only up to 2 cm maximum are sufficient for the treatment of locally advanced pancreatic cancer. These results are in accordance with most recent consensus guidelines, except that dorso-lateral margins, also with respect to sensitive OAR such as the kidneys, might be reduced based on our data [3].

Physical and technical means of dose-escalation may convert into an improvement of the therapeutic ratio. Considering that, modern techniques such as stereotactic body radiation therapy (SBRT), image guided radiation therapy (IGRT) or also particle therapy should be implemented in dose-escalation protocols. Preliminary data have shown excellent local control and low toxicity with single fraction of hypofractionated SBRT using different techniques, even in combination with chemotherapy [18-21]. However, in the future, more knowledge about organ motion will be acquired, and methods of compensation will be available; therefore, compensation of organ motion by separate target volumes such as internal target volumes (ITV) or elastic compensation methods such as tracking will be available in clinical routine.

## Conclusion

In conclusion, the present work demonstrates feasibility of a semi-automated system for recurrence analysis, which will be continuously optimized to eliminate manual steps in matching and segmentation. The system will be directly connected to an existing patient database, to provide a fully automated tool for fast analyses. Validation on the cohort of patients with pancreatic cancer demonstrated data well in accordance with previously published data, and could contribute to in-house optimization of subsequent study concepts based on an improved and validated target volume definition.

## Competing interests

There is no conflict of interest to report for this article.

## Authors’ contributions

KAK selected the data and performed the analysis, drafted and wrote the manuscript. DH segmented the recurrences and did the manual validation. AJ developed the registration and dose calculation tools. LZ developed the RTToolbox library. DH, JD and SEC were responsible for patient treatment and care. SEC contributed substantially to conception and design. DH, AJ, ROF and SEC reviewed/revised the manuscript. All authors read and approved the final manuscript.
